# Adult Gambling Problems and Histories of Mental Health and Substance Use: Findings from a Prospective Multi-Wave Australian Cohort Study

**DOI:** 10.3390/jcm10071406

**Published:** 2021-04-01

**Authors:** Stephanie S. Merkouris, Christopher J. Greenwood, George J. Youssef, Primrose Letcher, Suzanne Vassallo, Nicki A. Dowling, Craig A. Olsson

**Affiliations:** 1 Centre for Social and Early Emotional Development, School of Psychology, Faculty of Health, Deakin University, Geelong, VIC 3220, Australia; christopher.greenwood@deakin.edu.au (C.J.G.); george.youssef@deakin.edu.au (G.J.Y.); nicki.dowling@deakin.edu.au (N.A.D.); craig.olsson@deakin.edu.au (C.A.O.); 2 Centre for Adolescent Health, Murdoch Children’s Research Institute, Parkville, VIC 3052, Australia; pletcher@unimelb.edu.au; 3 Department of Pediatrics, University of Melbourne, Royal Children’s Hospital, Parkville, VIC 3052, Australia; 4 Australian Institute of Family Studies, Level 4, 40 City Road, Southbank, VIC 3006, Australia; suzanne.vassallo@aifs.gov.au; 5 Melbourne Graduate School of Education, University of Melbourne, Parkville, VIC 3053, Australia

**Keywords:** problem gambling, gambling, persistent, mental health, substance use, longitudinal, anxiety, depression, alcohol, tobacco, cannabis

## Abstract

Little is known about the cumulative effect of adolescent and young adult mental health difficulties and substance use problems on gambling behaviour in adulthood. We use data from one of Australia’s longest running studies of social and emotional development to examine the extent to which: (1) mental health symptoms (depressive and anxiety symptoms) and substance use (weekly binge drinking, tobacco, and cannabis use) from adolescence (13–18 years) into young adulthood (19–28 years) predict gambling problems in adulthood (31–32 years); and (2) risk relationships differ by sex. Analyses were based on responses from 1365 adolescent and young adult participants, spanning seven waves of data collection (1998–2014). Persistent adolescent to young adult binge drinking, tobacco use and cannabis use predicted gambling at age 31–32 years (OR = 2.30–3.42). Binge drinking and tobacco use in young adulthood also predicted gambling at age 31–32 years (OR = 2.04–2.54). Prior mental health symptoms were not associated with gambling and no risk relationships differed by sex. Findings suggest that gambling problems in adulthood may be related to the earlier development of other addictive behaviours, and that interventions targeting substance use from adolescence to young adulthood may confer additional gains in preventing later gambling behaviours.

## 1. Introduction

Gambling disorder is used in the Diagnostic and Statistical Manual of Mental Disorders (DSM-5) to define persistent and recurrent gambling behaviour that leads to clinically significant impairment or distress [1]. Problem gambling is a more general term that refers to gambling behaviour across a continuum of risk to the individual, families and friends, and the community [2]. Past-year Australian prevalence estimates, based on the Problem Gambling Severity Index [2], indicate that 0.4 to 0.6% of the population are classified as problem gamblers, with a further 1.9 to 3.7% and 3.0 to 7.7% classified as moderate-risk and low-risk gamblers, respectively [3,4]. While relatively low in prevalence, the burden of harm associated with gambling problems has been shown to be comparable to depression and alcohol use disorders [5]. Moreover, while problem gamblers experience more individual harms than moderate-risk and low-risk gamblers, 85% of the total burden of harm can be attributed to moderate and low-risk gamblers, due to their greater prevalence in the population [5]. These harms include a range of financial, relationship/interpersonal, emotional/psychological, health-related, cultural, educational/occupational and criminal outcomes [6].

In cross-sectional data, it has been well established that problem gambling co-occurs at a high rate with common mental health problems and substance use disorders [7,8]; specifically, nicotine dependence (56.4–60.1%), any mood disorder (23.1–37.9%), any anxiety disorder (17.6–37.4%), alcohol use disorder (21.2–28.1%), and illicit drug abuse/dependence (7.0–17.2%) [7,8]. Furthermore, people with gambling problems are over-represented in both mental health [9,10,11,12] and alcohol and other drug problem treatment settings [13,14]. Associations with mental health and substance use problems have also been reported early in development, in adolescence and young adulthood [15,16,17,18,19,20].

Findings from a recent meta-analysis of longitudinal data [21] examining child, adolescent, and young adult predictors of problem gambling suggest that depressive symptoms, alcohol use frequency, tobacco use, cannabis use, and illicit drug use increase risk for subsequent problem gambling severity, albeit with small effect sizes. In contrast, anxiety symptoms do not appear to predict the severity of problem gambling. However, there are few available studies and most longitudinal analyses have focused on single time-point exposures. Little is known about the influence of chronic exposure to risk factors spanning both adolescence and young adulthood (i.e., persistence), which can be more harmful than exposure to risk at a single point in time [22]. Moreover, in most longitudinal studies gambling outcomes have been assessed in either adolescence or young adulthood, with much less being known about longer terms outcomes in adulthood [21]. Finally, there have been few studies of sex-specific associations. Findings so far have suggested that mental health problems are more often associated with problem gambling in women [23,24,25,26,27,28] and hazardous alcohol use, cannabis use and tobacco use are more often associated with problem gambling in men [25,27,28,29,30]. Despite these sex differences, the limited evidence suggests that gender often fails to statistically moderate these risk relationships [26,31,32,33].

The purpose of this study was to address gaps in our understanding of the developmental relationship between mental health and substance use problems in adolescence and young adulthood, and later gambling problems in adult life. Specifically, the aims were to examine the extent to which: (1) mental health symptoms (depressive and anxiety symptoms) and substance use (binge drinking, tobacco use, and cannabis use) from adolescence (13–18 years) into young adulthood (19–28 years) predict gambling problems in adulthood (31–32 years); and (2) risk relationships differ by sex. Data were drawn from one of the Australia’s longest running studies of social and emotional development, which has followed a large cohort of families from infancy to adulthood (The Australian Temperament Project, est. 1983 [34]).

## 2. Methods

### 2.1. Participants and Procedure

Participants were from the Australian Temperament Project (ATP), a 16-wave longitudinal study tracking the psychosocial development of young people from infancy to adulthood. The baseline sample consisted of 2443 infants aged 4–8 months, recruited in 1983 from urban and rural areas and representative of the state of Victoria, Australia. Since then, families (parents from participant’s birth and also participants 11–12 years old onwards) have been invited to participate via mail surveys approximately every 2 years until 19–20 years of age and every 4 years thereafter. Further details regarding sample recruitment are provided elsewhere [34]. Data collection waves were variously approved by Human Research Ethics Committees at the University of Melbourne, the Australian Institute of Family Studies and/or the Royal Children’s Hospital, Melbourne.

To be included in the current study, participants needed to have provided relevant data in at least two of three developmental periods (i.e., adolescence ages: 13–14, 15–16, and 17–18 years; young adulthood ages: 19–20, 23–24, and 27–28 years; or adulthood age: 31–32 years). The resulting sample size was 1365 (738 women). Compared to the original 1983 sample, the current analytic sample had marginally lower rates of male participants, parents born overseas, and parents with high-school only education.

### 2.2. Measures

#### 2.2.1. Problem Gambling Severity 

Past-year problem gambling severity was assessed in adulthood (age 31–32 years) using the 9-item Problem Gambling Severity Index (PGSI) [2], as it is the preferred measure of problem gambling severity in population-level research [35,36,37,38]. Respondents rate on a scale from 0 ‘never’ to 3 ‘almost always’ how often they experience nine behavioural symptoms or consequences due to gambling (e.g., “Have you bet more than you could really afford to lose?”). Scores range 0–27, with higher scores indicative of greater problem gambling severity. These scores can be categorised into non-problem gambling (scores of 0), low-risk gambling (scores of 1–2), moderate-risk gambling (scores of 3–7) and problem gambling (scores of 8–27). In previous research, the PGSI has demonstrated high internal consistency, validity, sensitivity, and specificity [2]. PGSI scores had a strong positive skew and there were few people who endorsed risk (non-problem = 90%, low-risk = 6%, moderate-risk = 3%, problem = <1%). Given this, a binary variable was derived representing non-problem gambling (scores of 0) and any-risk gambling (scores of 1–27).

#### 2.2.2. Mental Health Symptoms 

Depressive and anxiety symptoms were self-reported by the participant using validated age-appropriate scales in adolescence and young adulthood.

Adolescent depressive symptoms were assessed using the 13-item Short Mood and Feelings Questionnaire [39,40]. Respondents were asked to rate their depressive symptoms, in the past 2 weeks, on a scale from 0 ‘not true’ to 2 ‘true’. At each assessment, the total score was summed and dichotomised at ≥11 to identify moderate to severe symptoms [41]. Adolescent anxiety symptoms were measured using adapted versions of the Child Behaviour Questionnaire (age 13–14 years; 5-items) [42] and Revised Children’s Manifest Anxiety Scale (ages 15–16 and 17–18 years; 11 items) [43]. On both scales, respondents rated how often they experienced anxious feelings on the same three-point scale from 0 ‘never/rarely’ to 2 ‘often/almost always’. Mean scores on both scales were dichotomised at >1 to identify moderate to severe symptoms.

Young adult depressive and anxiety symptoms were assessed using the Depression Anxiety and Stress Scales-Short Form (DASS-21) [44,45]. Participants rated their experience of depressive (seven items), anxiety (seven items) and stress symptoms (seven items) during the past week on a scale ranging from 0 ‘did not apply to me at all’ to 3 ‘applied to me very much or most of the time’. Given the correspondence between the DASS scales of stress and anxiety with generalised anxiety disorder and other anxiety disorders, respectively [45], both scales were used in unison to indicate anxiety symptoms. Total scores were dichotomised to identify moderate to severe symptoms of depression (≥7), anxiety (≥6), and stress (≥10), in accordance with the DASS manual [45].

#### 2.2.3. Substance Use Behaviours 

Frequency of binge drinking (≥5 drinks in either quick succession [ages 15–16 and 17–18 years] or during one day [ages 19–20, 23-24, and 27–28 years]), tobacco use, and cannabis use were all assessed as the number of days used in the past month at ages 15–16, 17–18, 19–20, 23–24, and 27–28 years. Tobacco use frequency was additionally assessed at age 13–14 years.

#### 2.2.4. Mental Health Symptom and Substance Use Behaviour Histories 

Binary variables were derived for each exposure to indicate the presence of any elevated mental health symptoms or weekly substance use (i.e., ≥4 days in the past month) in adolescence and young adulthood. Dichotomised variables were categorised into four history groups: ‘none’, ‘adolescence only’, ‘young adulthood only’ and ‘persistent’ (both adolescence and young adulthood).

#### 2.2.5. Potential Confounders 

Potential confounders included parent family background characteristics of country of birth (either parent not born in Australia), low parental education (< year 12) and separation/divorce during the participant’s childhood (ages 0–13 years). We also included participant sex, anti-social behaviour (two behaviours at least once or one behaviour more frequently; physical fights, damaged property, stolen, driven without permission, suspended or expelled, graffitied, carried a weapon, run away from home) across ages 13–18 years [46], parent-report of childhood behaviour problems (hyperactivity or hostile-aggressive mean scores ≥1 “applied somewhat”) across ages 11–13 years [42], and the age that participants reported they first started gambling (assessed retrospectively at age 31–32 years).

### 2.3. Statistical Analysis

All analyses were conducted in Stata 15 [47]. In a series of logistic regression analyses, the experience of any-risk gambling in adulthood was regressed onto each 4-level mental health and substance use history variable, in separate analyses. Analyses were conducted unadjusted and adjusted for confounders. Analyses were repeated including an interaction term between each mental health and substance use history and participant sex to explore sex-specific associations. Multiple imputation was used to manage missing data, for which missingness ranged 22–44% for exposures, 36% for the outcome, between 0–48% for potential confounding factors. Fifty complete datasets were imputed, based on a multivariate normal model [48]. Binary variables were imputed as continuous variables and then back transformed with adaptive rounding following imputation [49]. Estimates were obtained by pooling results across the 50 imputed datasets using Rubin’s rules [50]. Available case analyses were conducted to supplement the imputed data findings.

## 3. Results

Table 1 presents a summary of problem gambling severity, mental health and substance use histories, and potential confounding factors based on the imputed data. Eleven percent of the sample had experienced any-risk gambling during adulthood (non-imputed data: non-problem = 90%, low-risk = 6%, moderate-risk = 3%, problem = <1%). Mental health symptoms were common, with 58% reporting a history of elevated depressive symptoms during adolescence and/or young adulthood and 64% a history of elevated anxiety symptoms. A history of weekly binge drinking, tobacco, and cannabis use was experienced by 70%, 54%, and 27% of the sample, respectively. In comparison to the initial ATP sample of 2443, the current sample of 1365 evidenced some selective attrition of men and participants from families with low parental education and non-Australian backgrounds (Table S1).

**Figure 1:**
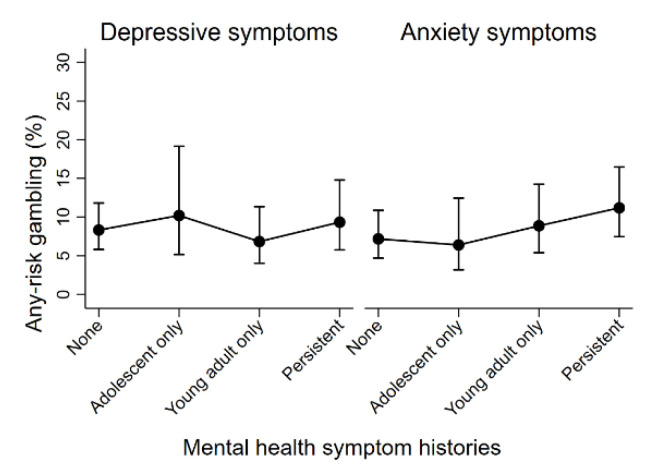
Estimated levels of any-risk gambling across mental health symptom historie.

**Figure 2:**
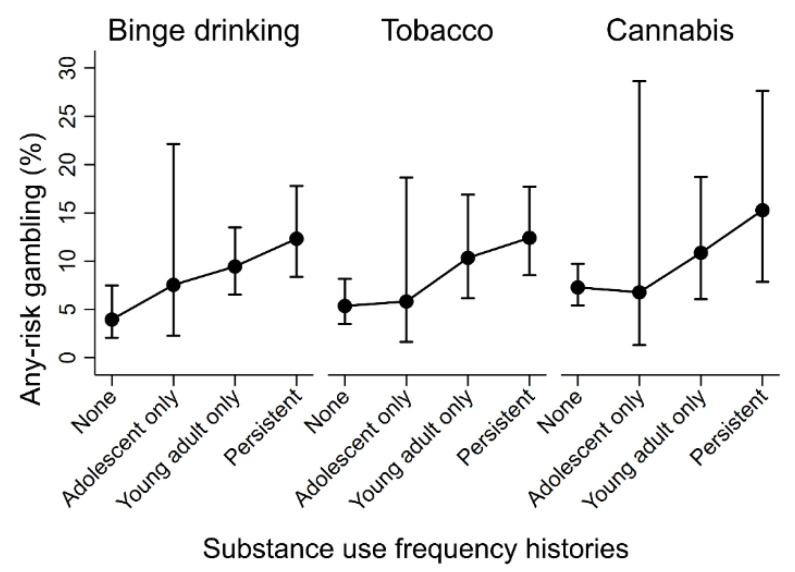
Estimated levels of any-risk gambling across substance use frequency histories.

Table 2 presents the results of analyses examining the relationships between mental health and substance use histories and any-risk gambling in adulthood, visualised in Figure 1 and Figure 2, respectively. For the mental health models, evidence did not support associations between the experience of any-risk gambling and histories of either elevated depressive or anxiety symptoms. For the substance use models, after adjustment for potential confounding factors, there was an increased odds of experiencing any-risk gambling in those with persistent histories of weekly binge drinking (OR = 3.42), tobacco use (OR = 2.50), and cannabis (OR = 2.30). To a lesser extent, there was also an increased odds of experiencing any-risk gambling in those with young adult only histories of weekly binge drinking (OR = 2.54) and tobacco use (OR = 2.04). Findings from the available case analyses were consistent with results from the imputed data and are presented in the Supplementary Material (Table S2).

In the interaction models associations between the experience of any-risk gambling and histories of elevated mental health symptoms (depressive *p* = 0.756; anxiety *p* = 0.524) or weekly substance use behaviours (binge drinking *p* = 0.964; tobacco *p* = 0.607; cannabis *p* = 0.867) were similar between men and women. These findings are supported by the strength and direction of associations between the experience of any-risk gambling and histories of mental health and substance use for men and women separately, which are presented in the Supplementary Material (Table S3).

## 4. Discussion

Findings from this study suggest that persisting substance use problems (adolescence into young adulthood), as well as substance use problems that begin in young adulthood, may play a role in the aetiology of gambling problems in adulthood. Persistent weekly substance use (all types) predicted gambling problems in adulthood, after accounting for a range of confounders including early externalising behaviour problems. Weekly binge drinking and tobacco use in young adulthood also predicted gambling problems in adulthood. Earlier mental health problems were not associated with gambling problems in adulthood. There was also no evidence of sex differences. These findings suggest that any-risk gambling in adulthood may be related to the early development and persistence of other addictive behaviours, for which interventions targeting substance use across both adolescence and young adulthood may confer benefits.

### 4.1. Substance Use Behaviours

The risk relationships we report between adolescent and young adulthood substance use and any-risk gambling in adulthood were notable [51]. This is particularly so given the extended time period over which risk was observed. Specifically, the odds of reporting any-risk gambling in adulthood were two- to three-fold higher in those with persisting histories of substance use problems than those without. Additionally, the odds of reporting any-risk gambling in adulthood were around two-fold higher in those reporting weekly binge drinking or tobacco use in young adulthood. Together, our findings suggests that gains in preventing adult problem gambling may be made from sustained investments in prevention of substance use problems from adolescence through to young adulthood [22].

Our findings extend on meta-analytic evidence by showing that persistent patterns of substance use pose greater risk for any-risk gambling in adulthood, compared to developmentally limited patterns of substance use [21]. Our findings further suggest that substance use behaviours may have a specific effect on any-risk gambling beyond that attributable to a general tendency for externalising behaviours, given the analytic adjustments for common causes including early antisocial and behavioural problems (hyperactivity and hostility-aggression). Importantly, our findings are also consistent with the numerous theories that have been proposed to explain the relationship between gambling problems and substance use disorders, such as the cross-substance coping response hypothesis (i.e., negative reinforcement promotes simultaneous use for self-regulation purposes, in which gambling can diminish the adverse effects of substance use and vice versa), the cross-substance cue reactivity model (i.e., due to repeated pairings, gambling and substance use cues acquire conditioned stimulus properties), the attention allocation model (i.e., alcohol myopia, in which substances impede on ones ability to process information and narrows ones attention to the most salient cues), and theories based on positive reinforcement principles (i.e., when substance use and gambling behaviours are engaged in concurrently, the positively rewarding effects are enhanced; or acute tolerance across both behaviours results in greater involvement in gambling as a way to provide alternative rewards) [52,53,54,55,56].

### 4.2. Mental Health Symptoms

We did not find evidence to suggest that common adolescent and young adult mental health problems predicted any-risk gambling in adulthood. This is consistent with meta-analytic evidence [21] that has similarly shown no prospective relationship between anxiety symptoms (at a single time-point) and subsequent gambling problems early in young adulthood. Moreover, while previous meta-analytic evidence has shown that depressive symptoms predict gambling problems, effect sizes were small and there was high between-study variability, in which many of the included studies found no significant association [21].

One explanation of our finding is that mental health symptoms may actually be consequences of gambling problems or may co-exist due to the sharing of common causes. This contrasts with other theorised pathways that posit gambling problems to be caused by pre-existing mental health symptoms, putatively due to gambling being used as a means to meet specific psychological needs [21,57]. The lack of association between mental health symptoms and gambling problems, however, may also be due to the binary categorisation of gambling problems, masking potential effects only visible with the full spectrum of risk. Future prospective research employing larger sample sizes across the continuum of risk is required in order to clarify the role of depressive and anxiety symptoms in the development of subsequent gambling problems.

### 4.3. Sex Differences

We found no evidence of sex differences. This is consistent with the limited number of studies that have likewise shown that sex did not moderate the relationship between gambling problems and mood, anxiety, alcohol use problems, nicotine dependence or substance use disorders [26,31,32,33], as well as a limited but increasing literature highlighting the lack of sex-specific patterns in the relationship between gambling problems, mental health symptoms and substance use [58]. These non-significant findings are also consistent with the gender-as-proxy hypothesis, which postulates that gender is not a direct risk factor, instead it acts as a proxy for factors that are commonly associated with it [26,59,60,61]. Our findings, therefore, support the notion that sex does not contribute to the prediction of any-risk gambling above and beyond other socio-demographic, gambling and psychological variables, such as those controlled for in our study [26].

### 4.4. Study Limitations

The findings we report need to be interpreted within the content of several sources of bias that are common in mature cohort studies [62]. While multiple imputation is used to minimise missing data bias, the retained sample differed from the original sample on measures of parental education and country of birth, and participant sex, reflecting typical trends in longitudinal studies. Relatedly, it is likely that there has been some selective drop-out of the most vulnerable individuals (e.g., those with early onset or significant gambling problems). This, in part, may have led to the necessary dichotomisation of gambling-related problems as none versus any, resulting in a limited ability to explore relationships with the continuum of gambling problems and reduced the overall power of the analyses to detect effects. The proportions of participants classified in each risk category, however, is consistent with national estimates [3,4] which suggest minimal effects of these sources of bias.

Furthermore, as data in the current study were collected using self-report measures, shared method variance remains a consideration, as does social desirability bias, given the sensitive nature of gambling problems [63]. Additionally, current measures do not allow for formal psychiatric diagnoses. Although confounder selection in the current study covered a range of demographic, contextual, and social factors, there remains a need to examine other factors that may confound the associations of interest, such as gambling-related factors (e.g., parental gambling problems) and impulsivity/compulsivity measures. Given that problem gambling severity was only evaluated at the last time-point, the complex bi-directional relationship between problem gambling and mental health symptoms and substance use behaviours could not be explored.

Taken together, future multi-wave longitudinal research employing larger samples of gamblers across the continuum of risk is needed. Whist non-clinical levels of gambling are associated with poor psychosocial functioning, future research could utilise semi-structured diagnostic interviews, so as to enable formal psychiatric diagnoses of these variables. Such research also needs to evaluate problem gambling severity at all time-points, to enable the evaluation of changes in problem gambling status over time, and the reciprocal relationship between persistent mental health symptoms and substance use variables and problem gambling status.

### 4.5. Implications

Findings from this study highlight the importance of substance use behaviours that have both persisted since adolescence and developed during young adulthood, in the development of gambling problems by adulthood. They also highlight the importance of continued investment in prevention of substance use problems at multiple and early stages across these critical developmental periods. Potential interventions could include school-based and family based interventions for adolescents that target a range of addictive behaviours, which have been shown to be effective for the prevention of later alcohol, tobacco and illicit substance use [64], as well as opportunistic delivery of brief screens and interventions for young adults [65] (e.g., general practice, university students). These findings also highlight the need for regular screening for gambling problems within alcohol and other drug treatment services, in order to identify at-risk individuals and provide appropriate resources and referrals [32,66]. It might also suggest the need for up-skilling alcohol and other drug treatment service providers in the delivery of brief and targeted interventions for individuals with co-occurring substance use and gambling problems [11].

## 5. Conclusions

The current multi-wave longitudinal study highlighted a potential role for binge drinking, tobacco use and alcohol use, but not depressive or anxiety symptoms, in the development of any-risk gambling in adulthood. Although both are important, substance use behaviours that had persisted since adolescence were associated with any-risk gambling to a greater extent than substance use behaviours which developed only during young adulthood. These findings suggest that any-risk gambling in adulthood may be related to the early development and persistence of other addictive behaviours, for which interventions targeting substance use across both adolescence and young adulthood may confer benefits. Prospective multi-wave longitudinal research, with larger samples including at-risk and problem gamblers, is needed to replicate these findings and gain a more in-depth understanding of the role of persistent mental health symptoms and substance use in the development of subsequent gambling problems.

## Figures and Tables

**Table 1:** Descriptive statistics (pooled) for analytic variables (*n* = 1365).

	*Problem Gambling Severity*		
	***n***	**%**	**95% CI**
Any-Risk Gambling	137	10.26	(8.24, 12.28)
	*Mental Health and Substance Use Histories*		
	***n***	**%**	**95% CI**
Depressive symptoms			
None	565	42.4	(39.34, 45.47)
Adolescence only	135	10.09	(8.17, 12.01)
Young adulthood only	307	23.01	(20.15, 25.86)
Persistent	327	24.5	(21.88, 27.12)
Anxiety symptoms			
None	483	36.24	(33.15, 39.33)
Adolescence only	222	16.66	(14.35, 18.97)
Young adulthood only	293	22.01	(19.18, 24.85)
Persistent	334	25.08	(22.44, 27.73)
Binge drinking			
None	394	29.55	(26.81, 32.3)
Adolescence only	60	4.48	(3.07, 5.89)
Young adulthood only	536	40.18	(37.16, 43.2)
Persistent	344	25.79	(23.04, 28.53)
Tobacco			
None	607	45.57	(42.52, 48.62)
Adolescence only	74	5.58	(4.04, 7.12)
Young adulthood only	223	16.74	(13.94, 19.54)
Persistent	428	32.11	(29.3, 34.91)
Cannabis			
None	977	73.3	(70.45, 76.15)
Adolescence only	43	3.25	(2.05, 4.45)
Young adulthood only	210	15.73	(13.16, 18.3)
Persistent	103	7.72	(6.09, 9.34)
	*Potential confounding factors*		
	***n***	**%**	**95% CI**
Parent non-Australian birth	360	26.98	(24.59, 29.37)
Parent separation/divorce	213	15.95	(13.98, 17.91)
Parent low education (<year 12)	342	25.69	(23.37, 28.01)
Women	721	54.07	(51.42, 56.71)
Adolescent anti-social behaviour	608	45.64	(42.95, 48.32)
Behaviour problems	267	20.04	(17.74, 22.35)
Early (<13 years) gambling	100	7.47	(5.52, 9.41)

**Table 2:** Models regressing any-risk gambling on to each mental health and substance use history.

	Unadjusted			Adjusted		
	OR	95% CI	p	OR	95% CI	p
Depressive symptoms						
None	1.00			1.00		
Adolescence only	1.13	(0.52, 2.46)	0.759	1.25	(0.56, 2.80)	0.583
Young adulthood only	1.06	(0.56, 2.01)	0.860	0.81	(0.42, 1.56)	0.524
Persistent	1.19	(0.67, 2.10)	0.560	1.13	(0.61, 2.09)	0.685
Anxiety symptoms						
None	1.00			1.00		
Adolescence only	0.75	(0.33, 1.72)	0.501	0.88	(0.37, 2.08)	0.775
Young adulthood only	1.57	(0.85, 2.91)	0.148	1.26	(0.67, 2.38)	0.478
Persistent	1.49	(0.81, 2.72)	0.196	1.63	(0.86, 3.08)	0.132
Binge drinking						
None	1.00			1.00		
Adolescence only	1.98	(0.48, 8.20)	0.343	1.98	(0.47, 8.37)	0.351
Young adulthood only	3.35	(1.62, 6.94)	0.001	2.54	(1.17, 5.50)	0.019
Persistent	5.09	(2.46, 10.53)	0.000	3.42	(1.54, 7.59)	0.003
Tobacco						
None	1.00			1.00		
Adolescence only	0.98	(0.24, 3.93)	0.978	1.09	(0.27, 4.49)	0.903
Young adulthood only	2.31	(1.18, 4.51)	0.014	2.04	(1.03, 4.05)	0.042
Persistent	2.71	(1.54, 4.77)	0.001	2.50	(1.34, 4.66)	0.004
Cannabis						
None	1.00			1.00		
Adolescence only	1.09	(0.20, 6.03)	0.924	0.93	(0.16, 5.36)	0.931
Young adulthood only	2.00	(1.04, 3.86)	0.038	1.55	(0.77, 3.14)	0.221
Persistent	3.19	(1.53, 6.61)	0.002	2.30	(1.01, 5.20)	0.046

Note: Each exposure run separately; Adjusted models controlling for parent country of birth, parent separation/divorce, parent low education, participant sex, participant adolescent antisocial behaviour, behaviour problems, and the age which participants reported they first started gambling.

## Data Availability

Ethics approvals for this study do not permit these potentially re-identifiable participant data to be made publicly available. Enquires about collaboration are possible through our institutional data access protocol: https://lifecourse.melbournechildrens.com/data-access/.

## Supplementary Materials

The following are available online at https://www.mdpi.com/article/10.3390/jcm10071406/s1, Table S1: ATP sample attrition, Table S2: Available case models regressing any-risk gambling on to each mental health and substance use history, Table S3: Models regressing any-risk gambling on to each mental health and substance use history in men and women.
