# Supplementary material for: Adult Gambling Problems and Histories of Mental Health and Substance Use: Findings from a Prospective Multi-Wave Australian Cohort Study
Source: J Clin Med. 2021 Apr 1;10(7):1406. doi: 10.3390/jcm10071406 (PMC8037618; doi:10.3390/jcm10071406)
Supplement: Supplementary file 1 [file jcm-10-01406-s001.pdf]

Supplementary Material

Table S1. ATP sample attrition

	Full ATP sample n = 2,443			Analytic sample n = 1,365			Differences
	N	n	%	N	n	%	p
G1 mother ≤High school	2384	1718	72	1361	906	67	<0.001
G1 father ≤High school	2331	1207	52	1350	632	47	<0.001
G1 mother not born in Australia	2407	479	20	1363	215	16	<0.001
G1 father not born in Australia	2378	634	27	1361	300	22	<0.001
G2 difficult temperament	2409	463	19	1363	243	18	0.199
G2 behaviour problems	2404	565	24	1362	310	23	0.520
G2 men	2433	1267	52	1365	627	46	<0.001

Table S2. Available case models regressing any-risk gambling on to each mental health and substance use history

	Unadjusted			Adjusted		
	OR	95% CI	p	OR	95% CI	p
Depressive symptoms						
None		1.00			1.00	
Adolescence only	0.81	(0.30, 2.20)	0.679	1.01	(0.34, 3.01)	0.982
Young adulthood only	0.71	(0.30, 1.70)	0.446	0.49	(0.19, 1.23)	0.129
Persistent	1.01	(0.48, 2.12)	0.987	1.12	(0.47, 2.66)	0.800
Anxiety symptoms						
None		1.00			1.00	
Adolescence only	0.54	(0.21, 1.39)	0.202	0.56	(0.19, 1.62)	0.280
Young adulthood only	1.47	(0.67, 3.21)	0.332	1.33	(0.55, 3.22)	0.527
Persistent	1.20	(0.56, 2.55)	0.635	1.85	(0.78, 4.41)	0.163
Binge drinking						
None		1.00			1.00	
Adolescence only	3.77	(0.69, 20.65)	0.126	2.70	(0.28, 25.99)	0.390
Young adulthood only	5.03	(1.87, 13.51)	0.001	3.97	(1.40, 11.30)	0.010
Persistent	7.38	(2.72, 20.02)	0.000	4.06	(1.37, 12.05)	0.011
Tobacco						
None		1.00			1.00	
Adolescence only	1.17	(0.25, 5.36)	0.844	1.61	(0.31, 8.26)	0.568
Young adulthood only	2.44	(0.98, 6.09)	0.055	2.85	(1.08, 7.49)	0.034
Persistent	3.62	(1.84, 7.13)	0.000	3.57	(1.61, 7.90)	0.002
Cannabis						
None		1.00			1.00	
Adolescence only		omitted			omitted	
Young adulthood only	2.48	(0.96, 6.42)	0.061	2.08	(0.73, 5.92)	0.169
Persistent	4.77	(2.04, 11.16)	0.000	3.66	(1.25, 10.73)	0.018

Note: Each exposure run separately; Adjusted models controlling for parent country of birth, parent separation/divorce, parent low education, participant sex, participant adolescent antisocial behaviour, behaviour problems, and the age which participants reported they first started gambling; omitted=adolescence only cannabis use omitted from analyses due to no cases of problem gambling .

Table S3. Models regressing any-risk gambling on to each mental health and substance use history in men and women

	Men			Women		
	OR	95% CI	p	OR	95% CI	p
Depressive symptoms						
None		1.00			1.00	
Adolescence only	1.07	(0.30, 3.80)	0.911	0.53	(0.09, 3.27)	0.491
Young adulthood only	1.48	(0.44, 4.99)	0.522	0.93	(0.41, 2.14)	0.866
Persistent	0.83	(0.40, 1.71)	0.614	1.52	(0.62, 3.72)	0.353
Anxiety symptoms						
None		1.00			1.00	
Adolescence only	0.93	(0.33, 2.59)	0.885	2.03	(0.49, 8.39)	0.327
Young adulthood only	1.01	(0.21, 4.83)	0.986	1.18	(0.51, 2.68)	0.700
Persistent	1.09	(0.55, 2.18)	0.801	3.03	(1.01, 9.04)	0.047
Binge drinking						
None		1.00			1.00	
Adolescence only	2.30	(0.25, 21.16)	0.463	2.09	(0.66, 6.56)	0.207
Young adulthood only	1.71	(0.24, 12.05)	0.591	4.16	(1.26, 13.68)	0.019
Persistent	3.17	(1.00, 10.00)	0.049	3.00	(0.96, 9.31)	0.058
Tobacco						
None		1.00			1.00	
Adolescence only	1.05	(0.18, 6.24)	0.954	2.53	(0.65, 9.87)	0.182
Young adulthood only	1.25	(0.13, 11.76)	0.845	1.98	(0.96, 4.06)	0.064
Persistent	1.87	(0.85, 4.12)	0.118	4.29	(1.47, 12.53)	0.008
Cannabis						
None		1.00			1.00	
Adolescence only	1.1	(0.18, 6.78)	0.919	1.57	(0.40, 6.20)	0.516
Young adulthood only	1.14	(0.16, 8.28)	0.896	2.09	(0.77, 5.69)	0.147
Persistent	1.49	(0.72, 3.07)	0.276	2.97	(0.91, 9.69)	0.072

Note: Each exposure run separately; Sex moderation models controlling for parent country of birth, parent separation/divorce, parent low education, participant adolescent antisocial behaviour, behaviour problems, and the age which participants reported they first started gambling; For models examining histories of depressive symptoms and cannabis use 1 and 10 of the imputed samples, respectively, had to be dropped to enable estimation.
